# Examining Clinical Symptoms and Personological Traits in Adolescents With Anorexia Nervosa: A Network Analysis Approach

**DOI:** 10.1002/jclp.70018

**Published:** 2025-07-19

**Authors:** Catherine Lessard, Alexandra Bédard, Elsa Rousseau, Dominique Meilleur, Caroline Pesant, Danielle Taddeo, Nathalie Gingras, Giuseppina Di Meglio, Pierre‐Olivier Nadeau, Richard Bélanger, Holly Agostino, Isabelle Thibault, Chantal Stheneur, Jean‐Yves Frappier, Catherine Bégin

**Affiliations:** ^1^ École de Psychologie Université Laval Québec QC Canada; ^2^ Centre Nutrition, Santé et Société (NUTRISS), Institut sur la Nutrition et les Aliments Fonctionnels (INAF) Université Laval Québec QC Canada; ^3^ Département d'Informatique et de Génie Logiciel, Faculté des Sciences et de Génie Université Laval Québec QC Canada; ^4^ Département de Psychologie Université de Montréal Montréal QC Canada; ^5^ Hôpital Fleurimont Centre Hospitalier Universitaire de Sherbrooke Sherbrooke QC Canada; ^6^ Centre Hospitalier Universitaire Sainte‐Justine Montréal QC Canada; ^7^ Centre de Pédopsychiatrie, Centre Intégré Universitaire de Santé et de Services Sociaux de la Capitale‐Nationale Québec QC Canada; ^8^ Département de Psychiatrie et de Neurosciences, Faculté de Médecine Université Laval Québec QC Canada; ^9^ Hôpital de Montréal pour Enfants/Montreal Children's Hospital, Centre Universitaire de Santé McGill Montréal QC Canada; ^10^ Centre Hospitalier Universitaire de Québec Québec QC Canada; ^11^ Département de Pédiatrie, Faculté de Médecine Université Laval Québec QC Canada; ^12^ Département de Psychoéducation Université de Sherbrooke Sherbrooke QC Canada; ^13^ Département de Pédiatrie, Faculté de Médecine Université de Montréal Montréal QC Canada

**Keywords:** adolescents, anorexia nervosa, anxiety, depressive symptoms, network analysis, personality

## Abstract

**Objective:**

Despite advancements in treatment, research indicates that fewer than half of patients with anorexia nervosa (AN) achieve full recovery by the end of their treatment. Network analysis provides a novel framework for understanding the central features of this complex psychopathology, potentially identifying critical elements that could enhance intervention strategies. While evidence suggests that personality traits play a significant role in the etiology, symptomatic expression, and maintenance of AN, no studies have yet utilized network analysis to explore the relationships among personality traits, eating disorder (ED) symptoms, and common psychiatric comorbidities in adolescents with AN. This study aims to fill this gap by applying network analysis to examine the interplay between ED symptoms, anxiety and depressive symptoms, and personality traits in a sample of 243 adolescents with AN (92.6% female), aged 12 to 17 years (M age = 14.7 ± 1.4), recruited from five University Health Centers across Quebec, Canada.

**Methods:**

Upon admission, participants completed the Eating Disorder Inventory‐3 (EDI‐3), the Multidimensional Anxiety Scale for Children (MASC), the Children's Depression Inventory 2 (CDI 2), and the Millon Adolescent Clinical Inventory (MACI). All variables were incorporated into a network analysis.

**Results:**

The analysis revealed that submissive, conforming, self‐demeaning, and inhibited personality traits were central nodes. Among ED symptoms, drive for thinness was the most prominent, with depressive symptoms exhibiting the highest centrality among comorbid conditions.

**Conclusion:**

These results highlight the complex interplay between ED symptoms, psychiatric comorbidities, and personality traits in adolescents with AN. These findings suggest that interventions may benefit from focusing on emotional regulation and interpersonal dynamics, with particular attention to assertiveness training and strategies to help adolescents express and address their needs in relationships.

## 1 Introduction

Anorexia nervosa (AN), an eating disorder (ED) typically onset during adolescence or early adulthood (American Psychiatric Association 2013), presents significant treatment challenges despite advancements in care. AN has the poorest prognosis among all ED diagnostic groups and is associated with a higher risk of mortality, with a peak age of death risk at 15 years (Miskovic‐Wheatley et al. 2023). Given the persistently low remission rates, there is a need to reassess our understanding of AN, considering its multifaceted nature, to improve treatment options for this population.

Classic nosography in psychiatry advances the existence of distinct diseases with specific underlying clinical symptoms that are then conceptualized as the manifestations of the mental condition itself. However, a growing body of research has focused on alternative models of conceiving psychological and behavioral symptoms in psychiatry, such as “psychological networks” using network analysis (Borsboom and Cramer 2013). According to network analysis theory, disorders do not emerge from a single latent cause but are rather characterized by dynamic networks of interacting, self‐reinforcing symptoms or clinical characteristics (Borsboom and Cramer 2013). It can then better fit the wide evidence of frequent comorbidities among psychiatric conditions that are not part of the psychopathology, but which can trigger symptoms (Borsboom 2002). This approach is of considerable relevance in AN, given the frequent comorbidity of mental disorders like anxiety and depression disorders, and the impact of such comorbidities on the outcome of AN (Arcelus et al. 2011; Fichter et al. 2006; Franko et al. 2018; Jagielska and Kacperska 2017; Wonderlich et al. 2005). Through this approach, it would be possible to elucidate interrelationships between AN and other mental disorder symptoms, highlight symptoms that may play an important role in the maintenance of AN, and use this information to guide both scientific research and clinical decisions on potential psychological intervention targets (Borsboom and Cramer 2013).

Some studies have applied network analysis to pediatric AN samples, either focusing solely on ED‐specific symptoms or, in some cases, also considering comorbid psychiatric symptoms such as anxiety and depression (Calugi et al. 2020; Goldschmidt et al. 2018; Monteleone et al. 2019). Goldschmidt et al. (2018) examined the interrelationships among ED symptoms using behavioral and attitudinal items from the Eating Disorder Examination (EDE; Fairburn and Cooper 1993) in a sample of 636 treatment‐seeking children and adolescents. Their study also sought to determine whether symptom networks differed across three ED diagnoses: AN, bulimia nervosa, and otherwise specified feeding and eating disorders (OSFED; Goldschmidt et al. 2018). Results from this study indicated that dietary restraint and concerns about weight and appearance emerged as central in the psychopathology across ED. Calugi et al. (2020) thereafter found similar findings in a sample of 547 treatment‐seeking adolescents with AN, observing that the most central and highly interconnected nodes in the network were related to shape overvaluation and desiring weight loss. Despite the importance of these findings, both studies have focused solely on ED symptoms, not considering the comorbid psychiatric symptoms (e.g., anxiety and depressive symptoms) that are highly related to AN, limiting the ability to assess the dynamic network between these comorbid psychiatric symptoms and those specific to ED in the pediatric population. However, among a sample of 405 hospitalized adolescents with a recent onset of AN, Monteleone et al. (2019) conducted network analysis to investigate the relationships between ED and comorbid psychiatric symptoms, namely anxious and depressive, posttraumatic stress, and obsessive‐compulsive symptoms. Results showed that depressive symptoms and personal alienation were the most central symptoms, followed by asceticism, posttraumatic stress problems, drive to thinness, low self‐esteem, and anxiety physical symptoms, suggesting their pertinence to be considered in treatments (Monteleone et al. 2019). These findings emphasized the importance of considering a broader spectrum of symptoms in network analysis to better conceptualize AN.

Interestingly, some personality traits have been associated with the etiology, symptomatic expression, and maintenance of AN (Cassin and von Ranson 2005; Gazzillo et al. 2013; Lavender et al. 2013; Podar et al. 1999; Turner et al. 2014; Westen and Harnden‐Fischer 2001; Wildes et al. 2011; Wonderlich et al. 2005). Previous research on personality traits in adolescents with ED has primarily relied on self‐reported measures using well‐established inventories such as the Millon Adolescent Clinical Inventory (MACI), Eating Disorder Inventory (EDI), and the Minnesota Multiphasic Personality Inventory (MMPI) (Dufresne et al. 2020). A meta‐analysis by Dufresne et al. (2020) aimed to synthesize findings on personality traits in adolescents with ED. The analysis reviewed studies assessing personality traits and categorized them based on DSM‐5 personality domains, identifying key personality dimensions that distinguish adolescents with ED from their non‐ED counterparts. Results indicated that adolescents with ED exhibited higher levels of traits within three DSM‐5 personality domains: conscientiousness‐related traits (e.g., persistence, asceticism, overcontrol, perfectionism), negative affectivity (e.g., neuroticism, depressive affect, emotional dysregulation), and detachment (e.g., inhibition, introversion, social alienation, lower novelty‐seeking) (Dufresne et al. 2020). Notably, the presence of an AN diagnosis moderated these relationships, showing a stronger association with conscientious traits than other ED. These findings highlight a complex interplay between ED symptoms and personality traits. However, no study to date has integrated personality traits into a network analysis incorporating ED‐specific behaviors and comorbid psychiatric symptoms, underscoring the need for further research, particularly in pediatric AN populations.

The objective of the present study was to apply the network analysis approach in a sample of adolescents with AN to describe the interconnections between AN specific symptoms, comorbid psychiatric symptoms (i.e., anxiety and depressive symptoms), and personality traits. The network analysis allowed for the identification of central symptoms in AN that could be considered in AN treatment. Based on previous network analysis studies, we hypothesized that desire for thinness and body dissatisfaction, as well as depressive symptoms, would be central symptoms in the network. Moreover, given that adolescents with ED are more likely to exhibit traits associated with conscientiousness, negative affectivity, and detachment than adolescents without ED (Dufresne et al. 2020), we anticipated that traits associated with these domains would also emerge as central nodes in the network.

## 2 Methods

### 2.1 Participants

Adolescents aged between 12 and 17 years were recruited upon their entry into the specialized ED services (inpatient and outpatient treatment) at five University Health Centers across the Province of Quebec, in Canada. These five centers included: (1) the Douglas Mental Health University Institute, (2) the Montreal Children's Hospital ‐ McGill University Health Center, (3) the Fleurimont Hospital (CIUSSS de l'Estrie‐CHUS), (4) the Centre de pédopsychiatrie de Québec (CIUSSS de la Capitale‐Nationale) and (5) the CHU Sainte‐Justine. To be included in the study, a diagnosis of AN (typical or atypical AN) according to the DSM‐5 criteria (American Psychiatric Association 2013) had to be confirmed by the attending physician (i.e., pediatricians or psychiatrists) specialized in ED.

### 2.2 Procedure

Upon entering the service and receiving a confirmed AN diagnosis through an interview with the attending physician, patients were sent an electronic link to complete a self‐report assessment. This self‐report assessment collected data on the patient's ED symptoms and psychological traits, anxiety symptoms, depressive symptoms, and personality traits, among others. The medical team also collected medical data. Before participating in this study, all adolescents provided their written informed assent, in addition to their parents/legal guardians written informed consent. This study has been approved by the ethics committee of the coordinating center (CHU de Québec‐Université Laval: # MP‐20‐2015‐2323) and by the Ethic Committees of each participating center.

#### 2.2.1 Demographic and Medical Characteristics

Demographic data, including age, gender, ethnicity, were reported by the patients and/or their parents/legal guardians. Medical data, such as diagnosis (restrictive AN [AN‐R] or binge‐eating/purging AN [AN‐BP]), body mass index (BMI) and z‐BMI, and patient treatment (i.e., inpatient or outpatient), were reported by the medical team.

### 2.3 Variables Included in the Network Analysis

#### 2.3.1 ED Symptoms and Psychological Traits

The EDI‐3 (Garner 2004) is a self‐reported questionnaire used to assess symptoms and psychological traits relevant to ED. The questionnaire consists of 91 items that are scored using a six‐point Likert scale that ranges from never to always. For each statement, participants were asked to decide whether it describes them ‘always’, ‘usually’, ‘often’, ‘sometimes’, ‘rarely’, or ‘never’. After reverse‐scoring the negatively keyed items, total raw scores for each subscale were obtained by adding the relevant items (Garner 2004). The twelve raw subscales (i.e., drive for thinness, bulimia, body dissatisfaction, low self‐esteem, personal alienation, interpersonal insecurity, interpersonal alienation, interoceptive deficits, emotional dysregulation, perfectionism, asceticism, and maturity fears) were used in the present study. Higher scores indicate a higher severity. Cronbach's alphas in the present study were as follows: 0.91 (Drive for thinness), 0.84 (Bulimia), 0.91 (Body dissatisfaction), 0.88 (Low self‐esteem), 0.91 (Personal alienation), 0.82 (Interpersonal insecurity), 0.80 (Interpersonal alienation), 0.88 (Interoceptive deficits), 0.82 (Emotional dysregulation), 0.78 (Perfectionism), 0.79 (Asceticism) and 0.79 (Maturity fear).

#### 2.3.2 Anxiety Symptoms

The presence of symptoms related to anxiety disorders in adolescents was assessed using the MASC (March 1997). This tool includes four subscales: (1) physical symptoms, (2) harm avoidance, (3) social anxiety, and (4) separation/panic anxiety. This self‐report questionnaire consists of 39 questions answered on a four‐point Likert scale ranging from ‘never true about me’ to ‘often true about me’. Participants were asked to refer to symptoms experienced ‘recently’ (i.e., ‘This questionnaire asks about what you have thought, felt, or how you have acted ‘recently’). In addition to the four subscales, we calculated a global severity score for anxiety symptoms by summing all 39 items, and used this total raw score in the analysis. Higher scores indicate a higher severity. In this study, internal consistency for the global severity scale was 0.90.

#### 2.3.3 Depressive Symptoms

Emotional, cognitive, and behavioral symptoms of depression in adolescents over the past 2 weeks were assessed using the CDI 2 (Kovacs 2011). This self‐report questionnaire consists of 28 questions pertaining to four subscales: (1) negative mood/physical symptoms, (2) negative self‐esteem, (3) ineffectiveness, and (4) interpersonal problems. The questions are answered on a three‐point Likert scale ranging from no depressive symptoms to significant depressive symptoms. After reverse‐scoring the negatively keyed items, the scores were summed to obtain an overall total score for depressive symptoms. In the present study, we only used the total score, with a higher score indicating a greater possibility for the child to experience depressive symptoms. Internal consistency for the total score was 0.92 in the present study.

#### 2.3.4 Personality Traits

Personality traits were assessed using the MACI (Millon et al. 1993, 2006). This self‐reported questionnaire consists of 160 true or false questions and includes 27 scales grouped under three categories: (1) personality patterns, (2) clinical syndromes, and (3) expressed concerns. For the present study, the 12 ‘personality patterns’ raw scores were used (i.e., introversive, inhibited, doleful, submissive, dramatizing, egotistic, unruly, forceful, conforming, oppositional, self‐demeaning, and borderline tendencies). Higher scores indicate more pathological traits. In the present study, the Cronbach alphas were as follows: 0.88 (Introversive), 0.90 (Inhibited), 0.92 (Doleful), 0.60 (Submissive), 0.67 (Dramatizing), 0.72 (Egotistic), 0.79 (Unruly), 0.78 (Forceful), 0.64 (Conforming), 0.87 (Oppositional), 0.94 (Self‐demeaning), and 0.84 (Borderline tendencies).

### 2.4 Data Preparation

#### 2.4.1 Preliminary Steps

Participants with only missing data were excluded from the analyses. We examined the mean and standard deviation, skewness, and kurtosis. Because the test revealed a nonnormal distribution for many of the variables, we used nonparanormal transformation through the R‐package huge version 1.3.5, as advised (Epskamp et al. 2016).

#### 2.4.2 Nodes Selection (I.E., “Key Symptoms or Variables”)

If several items are too similar in content (i.e., if items within a scale have extremely high average‐interitem correlations), it may bias centrality estimates by artificially inflating strength centrality (Levinson et al. 2018). As the inclusion of multiple nodes evaluating the same construct could lead to misinterpretation of the network analysis, selecting the right variables is therefore crucial.

To ensure accurate results, we included only the CDI 2 total score, avoiding the inflation of strength centrality. Indeed, based on the findings from previous studies, it appears that a single higher‐order factor could potentially be the most appropriate way to represent the structure of the CDI 2 (Lee et al. 2012). This is supported by research that utilized discriminant analyses, revealing that accurate classification of adolescents diagnosed with major depression was achieved when using either the total CDI 2 score (i.e., ignoring factor structure) or considering a higher‐order factor model (Lee et al. 2012).

Secondly, we conducted an analysis to determine whether the subscales used in the network analysis capture distinct symptoms, or if their correlations with other subscales indicate excessive overlap. To perform this analysis, the goldbricker function from the R networktools package was used (Jones 2020). This function compares the correlation of one node with each item in the data set to the correlation of another node with each item in the data set, and so forth, for every item. This leads to a proportion of correlations, and if it is significantly different from the others, it is considered that the two nodes measure different symptoms. On the other hand, if the proportion of correlations is not significantly different, it can be assumed that it does not add valuable information and could lead to inflating strength centrality. Based on previous literature, a cut‐off point of 0.25 was selected for this significant proportion, with a p‐value threshold of 0.05 (Levinson et al. 2018). The net_reduce function was subsequently applied to combine the suggested reductions through principal component analysis if they were deemed clinically appropriate. Accordingly, four ‘bad pairs‘ were identified using the goldbricker function and analyzed by a psychologist (CB) and a doctoral candidate in psychology (CL):

1. MACI Doleful and Self‐Demeaning: Both dimensions emphasize pain and a loss of joy/pleasure, which can impact interpersonal interactions (e.g., restricting emotional experience and expression in relationships). These commonalities justify their combination from a clinical perspective.
2. EDI‐3 Asceticism and Interoceptive Deficits: No commonalities justify their combination from a clinical perspective, as they address distinct psychological mechanisms. Asceticism emphasizes self‐imposed restrictions and a tendency to suppress or control bodily needs (such as hunger) through behaviors like fasting or excessive exercise. This reflects a mindset focused on rigid discipline aimed at bodily control. In contrast, Interoceptive Deficits relate to difficulties in accurately recognizing emotional states.
3. MASC Physical Symptoms and EDI‐3 Interoceptive Deficits: No commonalities justify their combination from a clinical perspective, as MASC physical symptoms reflect anxiety‐related somatic complaints, whereas EDI‐3 interoceptive deficits denote difficulties in accurately recognizing emotional states.
4. MASC Physical Symptoms and EDI‐3 Asceticism: No commonalities justify their combination from a clinical perspective. MASC physical symptoms reflect anxiety‐related somatic complaints, whereas EDI‐3 asceticism emphasizes self‐imposed restrictions and a tendency to suppress or control bodily needs (such as hunger) through behaviors like fasting or excessive exercise. This reflects a mindset focused on rigid discipline aimed at bodily control.

As a result, only the nodes MACI self‐demeaning and doleful were combined using the net_reduce function. Because EDI‐3 asceticism and interoceptive deficits are theoretically distinct from each other, we maintained both items as separate nodes. As the physical symptoms of the MASC exhibited considerable overlap with other nodes, we opted to utilize only the MASC total scale in the current study. Using the total score helped mitigate the issue of ‘bad pairs’. Thus, the final network comprised 25 variables including all 12 subscales of the EDI‐3, the total score of the MASC, the total score of the CDI 2 and eleven subscales of the MACI, including the self‐demeaning and doleful node combined.

## 3 Network Analysis

According to the methods described in Epskamp et al. (2016), network analysis was performed using *qgraph* package. R codes are provided in the Supporting Information. We estimated the network structure using the Gaussian Graphical Model (Epskamp et al. 2016). In this model, the connections (i.e., edges) represent the unique association among two variables, after accounting for the impact of all other variables in the network. Therefore, each connection in the network reflects a partial correlation coefficient between two variables (Epskamp and Fried 2018). In the present study, continuous variables were used. Network relationships do not indicate directionality, which means that there is no inference of causation or direction in the relationship. To address the issue of spurious connections and retain only meaningful associations, we applied a regularization using the ‘least absolute shrinkage and selection operator‘ and Extended Bayesian Information Criterion (EBIC) model selection, which shrinks small partial correlations, setting them to zero, so only the most robust partial correlations remain visible (Epskamp and Fried 2018). The EBIC, a parameter that sets the degree of regularization/penalty applied to sparse correlations, was set to 0.5 in the present analysis (Foygel Barber and Drton 2015). Variables missing at random were dealt with by using pairwise complete observations (i.e., participants were not deleted listwise, but rather all available information was used to estimate each correlation).

The indicators of centrality were calculated using the *centralityPlot* function in *qgraph* (i.e., strength, betweenness, and closeness). The total absolute edge‐weights between a node and every other node to which it is connected in the network are referred to as the node *strength*. *Strength* is highly important for psychopathology because it is assumed that the activation of a node (i.e., symptom) with high strength centrality will lead to the activation of other nodes (McNally 2016). Most clinical researchers rely on *strength* centrality when interpreting psychopathology networks, partially because it tends to be more stable (Levinson et al. 2018) and more interpretable than *betweenness* and *closeness* (Forbes et al. 2017). The relevance of a node in terms of its connection to other nodes is described by its node *betweenness*, while the average distance between a node and every other node in the network is referred to as the node *closeness* (Epskamp and Fried 2018).

To assess the internal reliability of the network according to Epskamp et al.‘s recommendations (2017), bootstrapped confidence intervals were initially computed using ‘nonparametric‘ bootstrapping (nboots 2500) to gauge the precision of centrality indices via the bootnet function. Subsequently, the correlation stability coefficient was calculated to determine the maximum percentage of the population that could be eliminated while maintaining a correlation of at least 0.7 between the recalculated indices of the derived networks and the original network (Epskamp et al. 2017). Network stability analyses were also conducted using the bootnet package (Epskamp et al. 2017). The correlation stability coefficient was further assessed to evaluate the stability of centrality indices, where networks with reliable centrality should exhibit a correlation stability coefficient greater than or equal to 0.25, ideally surpassing 0.5 for centrality estimates (Epskamp et al. 2017).

## 4 Results

### 4.1 Participant Characteristics

Two hundred and forty‐three patients aged between 12 and 17 years old were included in the present analyses. Females represented 92.6% (*n* = 225) of the sample. Participants were 14.7 ± 1.4 years old on average. For the DSM‐5 diagnosis, 84.0% of patients were diagnosed with AN‐R and 11.5% with AN‐BP (4.5% of missing data). Demographic and clinical characteristics of the sample are presented in Table 1.

**Table 1:** Demographic and clinical characteristics of the sample.

	Mean ± SD	Percent
**Gender**		
Male		7.4
Female		92.6
**Age**	14.7 ± 1.4	
**BMI (kg/m^2^)**	17.6 ± 2.7	
**z‐BMI**	−1.16 ± 1.13	
**Diagnosis**		
AN‐R		84.0
AN‐BP		11.5
Missing		4.5
**Treatment at admission**		
Inpatient		23.5
Outpatient		66.7
Missing		9.8
**Ethnicity**		
Caucasian		84.0
Asian		3.3
Latin		1.6
African		1.6
Other		3.3
Missing		6.2

*Note:* Because there were some missing values, the number of participants ranged from 219 to 243.

Abbreviations: AN‐BP, Binge‐eating/purging anorexia nervosa; AN‐R, restrictive anorexia nervosa; BMI, Body mass index.

### 4.2 Network Analysis

**Figure 1:**
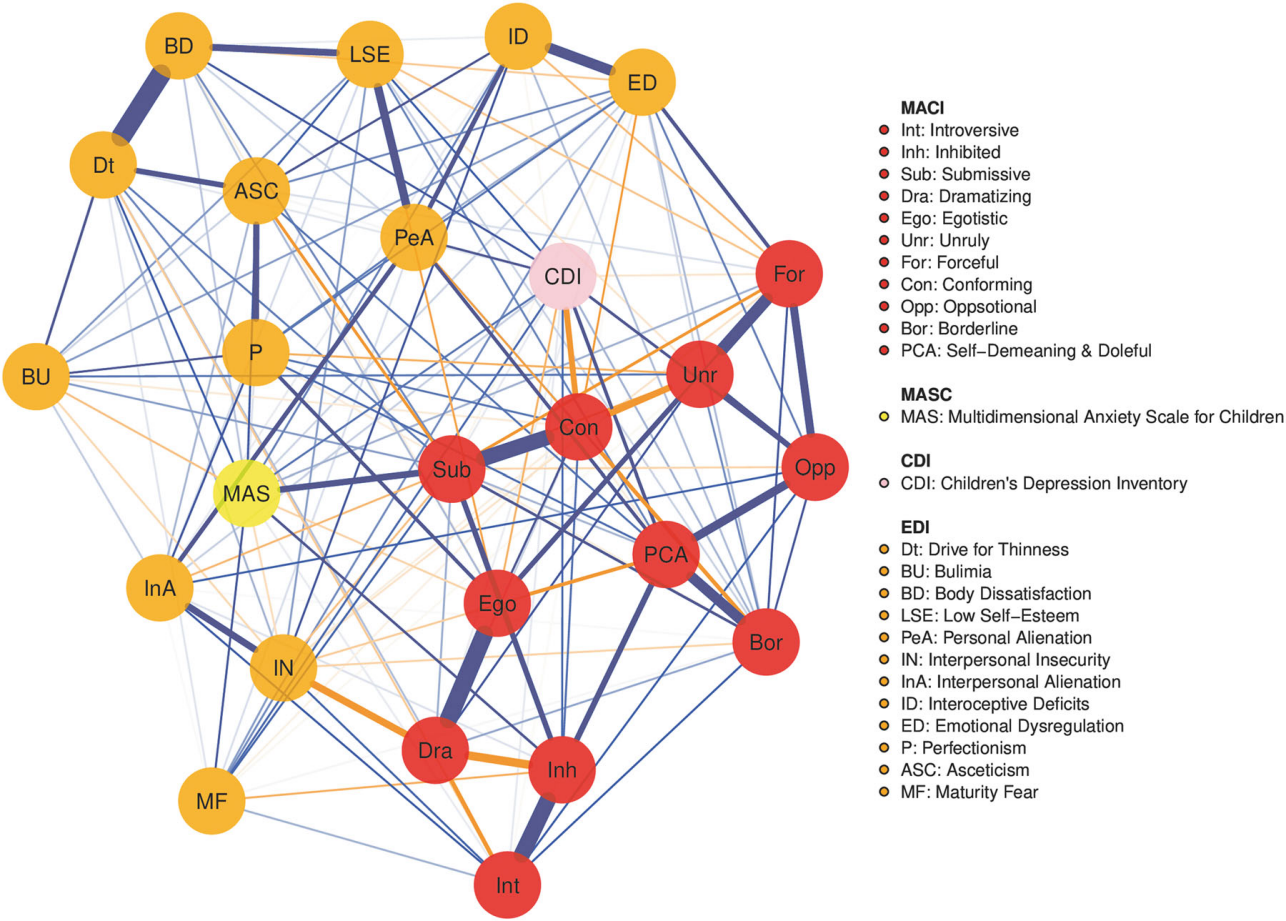
Network structure comprising the EDI‐3 subscores, the MASC total score, the CDI 2 total score, and the MACI subscores. Thicker edges (i.e., line connections between nodes) represent stronger relationships. Blue edges indicate positive correlations while orange edges illustrate negative correlations.

**Figure 2:**
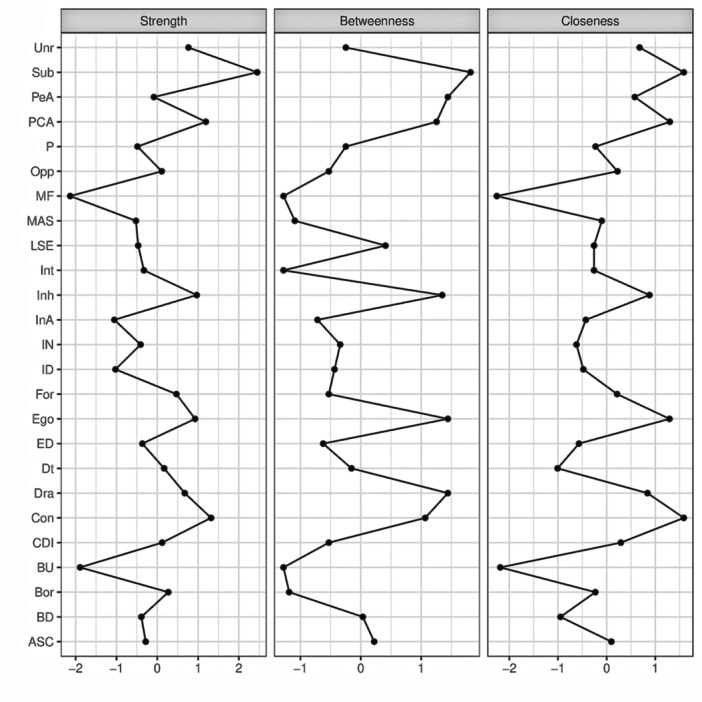
Plot of centrality indices of the included variables. ASC, asceticism; BD, body dissatisfaction; Bor, borderline; BU, bulimia; CDI, children's depression inventory; Con, conforming; Dra, dramatizing; DT, drive for thinness; ED, emotional dysregulation; Ego, Egotistic; For, forceful; ID, interoceptive deficits; IN, interpersonal insecurity; InA, interpersonal alienation; Inh, inhibited; Int, introversive; LSE, low self‐esteem; MAS, Multidimensional Anxiety Scale for Children; MF, maturity fear; Opp, oppositional; P, perfectionism; PCA, self‐demeaning and doleful; PeA, personal alienation; Sub, submissive; Unr, unruly.

Figure 1 shows the network structure comprising the EDI‐3 subscales, the MASC total scale, the CDI 2 total scale, and the MACI subscales. Blue edges indicate positive correlations, while orange edges illustrate negative correlations. The plot of centrality indices for the included variables is presented in Figure 2. Centrality indices estimates are reported in Table S1. The accuracy of centrality indices is shown in Figure S1, while the bootstrapped confidence intervals are reported in Figure S2.

The correlation stability coefficient of the network was 0.671 for *strength*, 0.519 for *betweenness*, and 0.671 for *closeness*. *Strength* and *closeness* emerged as the centrality index with the highest stability. *Strength* is highly important for psychopathology because it is assumed that the activation of a node (i.e., symptom) with high strength centrality will lead to the activation of other nodes (McNally 2016). Given that the literature suggests an unclear interpretation of *betweenness* and *closeness* in networks (Forbes et al. 2017), only the *strength* was used to describe our results. *Strength* centrality indices are reported in Figure 2 and Table S1. Nodes with the highest *strength* centrality were submissive (*M* = 2.010), conforming (*M* = 1.666), the combined self‐demeaning and doleful (*M* = 1.627), and inhibited (*M* = 1.559). Among EDI symptoms, drive for thinness exhibited the highest centrality (*M* = 1.315), followed by personal alienation (*M* = 1.239), asceticism (*M* = 1.178), and emotional dysregulation (*M* = 1.152). For the comorbid symptoms, the depressive symptoms total score (*M* = 1.300) demonstrated greater strength centrality compared to the anxiety symptoms total score (*M* = 1.105).

The correlation matrix is provided in Table S2. The submissive node exhibited the strongest correlations with conforming (*r* = 0.522), anxiety symptoms (*r* = 0.405), and inhibited (*r* = 0.403) nodes. The conforming node demonstrated the strongest correlations with submissive (*r* = 0.522), egotistic (*r* = 0.339), and dramatizing (*r* = 0.317) nodes. The combined self‐demeaning and doleful node displayed stronger correlations with depressive symptoms (*r* = 0.884), borderline node (*r* = 0.872), and personal alienation (*r* = 0.853) nodes. The inhibited node exhibited strong correlations with introversive (*r* = 0.908), combined doleful and self‐demeaning (*r* = 0.824), and depressive symptoms (*r* = 0.765) nodes.

When examining the most central nodes of the EDI, drive for thinness exhibited stronger correlations with body dissatisfaction (*r* = 0.809), asceticism (*r* = 0.615), and low self‐esteem (*r* = 0.601) nodes. The personal alienation node showed strong correlations with combined self‐demeaning and doleful (*r* = 0.853), depressive symptoms (*r* = 0.836), and low self‐esteem (*r* = 0.811) nodes. The asceticism node displayed the strongest correlations with combined self‐demeaning and doleful (*r* = 0.656), personal alienation (*r* = 0.651), and low self‐esteem (*r* = 0.630) nodes. The emotional dysregulation node showed stronger correlations with oppositional (*r* = 0.669), interoceptive deficits (*r* = 0.641), and borderline (*r* = 0.630) nodes.

Finally, the depressive symptoms node exhibited stronger correlations with combined self‐demeaning and doleful (*r* = 0.884), personal alienation (*r* = 0.836), and borderline (*r* = 0.803) nodes.

## 5 Discussion

This study represents the first attempt to use the network analysis approach to explore the interconnections between ED specific symptoms, comorbid symptoms (i.e., anxiety and depressive symptoms), and personality traits in a pediatric AN sample. Compared to previous studies, we encompassed, in our network analysis, a broader array of features, including personality traits, to achieve a deeper understanding of the patterns of interrelationships among symptoms and traits at intake to specialized ED services. Based on findings from previous studies (Calugi et al. 2020; Goldschmidt et al. 2018; Monteleone et al. 2019), we hypothesized that the desire for thinness, body dissatisfaction, and depressive symptoms would emerge as central symptoms in the network. Additionally, we anticipated that some traits associated with three of the five personality domains of the DSM‐5, namely conscientiousness, negative affectivity and detachment, would be central in our network analysis, considering that adolescents with ED present a greater propensity for these traits than those without ED (Dufresne et al. 2020). Our results are consistent with the stated hypotheses, demonstrating that the desire for thinness is central to the clinical portrait of our sample of adolescents with AN. Contrary to the hypothesis, however, body dissatisfaction was not a central node in our sample; nevertheless, it was strongly associated with the desire for thinness. As expected, depressive symptoms also emerged as central comorbid psychiatric features in the clinical presentation of adolescents with AN, supporting their prominent role in the disorder's psychopathology. In addition, traits related to conscientiousness (i.e., asceticism), negative affectivity (i.e., submissiveness, conformism, emotional dysregulation), and detachment (i.e., self‐demeaning and doleful traits, inhibition, personal alienation) were also central in our network. Overall, the network analysis highlighted the complex interplay between ED specific symptoms, comorbid psychiatric symptoms, and personality traits in adolescents with AN. Our results support those of the literature and represent an interesting step towards the use of an integrative conceptualization of personality in the field of ED.

The analysis of ED‐core symptoms revealed that the node which was the most central in the network was drive for thinness. Drive for thinness, which refers to an extreme desire to be thinner, a concern with dieting, a preoccupation with weight, and an intense fear of weight gain, is recognized as a core feature of ED and a crucial diagnostic criterion across many classification systems (Garner 2004). This finding is consistent with those from previous network analysis studies in both adolescent (Calugi et al. 2020; Goldschmidt et al. 2018; Monteleone et al. 2019) and adult patients with AN (Elliott et al. 2020; Solmi et al. 2019). Although previous studies have also highlighted body dissatisfaction as a central feature of ED (Calugi et al. 2020; Goldschmidt et al. 2018), our network analysis did not identify it as a central node. However, its strong association with the drive for thinness reinforces its critical role in the treatment of AN, suggesting that it remains a key factor that should not be overlooked. Depressive symptoms have also been identified as central in our network analysis. This finding is consistent with previous studies, indicating that individuals of all ages with AN tend to exhibit a clinical profile marked by higher levels of depressive symptoms compared to controls (Brand‐Gothelf et al. 2014; Bühren et al. 2014; Farstad et al. 2016). It is also in line with previous studies employing network analysis methods, identifying depressive symptoms as highly central in AN samples (Monteleone et al. 2019; Solmi et al. 2018, 2019).

In addition to these symptoms, asceticism, a trait associated with the DSM‐5 conscientiousness domain (Dufresne et al. 2020), was also identified as a central node in our network. Asceticism refers to the pursuit of virtue through spiritual ideals such as self‐discipline, self‐denial, and control of bodily urges (Garner 2004). Our finding aligns with a network analysis conducted by Monteleone et al. (2019) in adolescents with AN, which also identified asceticism as a central node. This is also consistent with a previous meta‐analysis that found a strong association between AN and conscientious traits, such as asceticism (Dufresne et al. 2020). Previous research has suggested that certain personality traits exhibit stronger associations with specific AN subtypes (Farstad et al. 2016). For instance, it is interesting to highlight that studies have shown that individuals with AN‐R, representing 84% of our sample, score higher on conscientious traits than those with bulimia nervosa (BN) and AN‐BP (Bollen and Wojciechowski 2004; Tasca et al. 2009), suggesting that these traits may modify the clinical presentation of AN in adolescence, by increasing the vulnerability to restrict. Our network analysis also reveals a proximity between the nodes drive for thinness and asceticism. This interconnection between these two nodes is consistent with theoretical frameworks that view asceticism not only as a standalone trait but also as a driving force behind the drive for thinness (Garner and Bemis 1982, 1985; Vitousek and Ewald 1993; Vitousek and Hollon 1990). It suggests that the pursuit of thinness might be fueled by an underlying ascetic motivation, where self‐denial and control over bodily urges become central to the individual's approach to weight loss (Garner and Bemis 1982, 1985; Vitousek and Ewald 1993; Vitousek and Hollon 1990). Incorporating an understanding of this relationship into clinical practice could enhance treatment approaches by addressing both the drive for thinness and the ascetic motivations that may underpin it.

Submissive, conforming, and emotional dysregulation traits, some features that can be related to the DSM‐5 negative affectivity domain, have also been identified as central in our network analysis. The present result aligns with those of Dufresne et al. (2020), whose meta‐analysis demonstrated an elevated presence of negative affectivity in adolescents with ED compared to the general adolescent population. Negative affectivity is characterized by frequent and intense experiences of a wide range of negative emotions, along with their behavioral and interpersonal manifestations (American Psychiatric Association 2013). According to the classical model of personality and negative affectivity (American Psychiatric Association 2013), traits such as emotional instability and a heightened sensitivity to negative emotions may lead to self‐harming behaviors. In the context of AN, behaviors such as food restriction or purging can serve to manage or numb these negative emotions. Additionally, individuals with AN may engage in these behaviors to elicit care, attention, or emotional support from others, without directly naming their needs. In this sense, AN's symptoms may function as a means of reinforcing close relationships and seeking reassurance or emotional validation. As such, conforming is negatively related to depressive symptoms as if complying gives patients the impression that they are doing better. However, it does not allow them to connect with their actual needs. These findings suggest that interventions should include a better awareness of the emotional needs underlying anorexic behaviors, as well as the implementation of exercises and role‐playing to assist patients in expressing their needs, setting boundaries, and reducing interpersonal dependency. These interventions will help enhance the patient ‘s sense of autonomy.

Our results revealed that traits related to detachment, namely self‐demeaning and doleful traits, inhibition, and personal alienation, were also central nodes in our pediatric AN sample. Our results are consistent with a previous network analysis in adolescents with AN, identifying personal alienation as a central node (Monteleone et al. 2019), as well as with Dufresne et al.‘s (2020) meta‐analysis, which highlighted an increased risk of detachment in individuals with ED compared to those without. Interpersonal mistrust, social withdrawal, and restricted emotional expression, characteristics associated with detachment, can hinder their ability to form meaningful relationships despite an underlying need for social connection (Dufresne et al. 2020; Stuart and Robertson 2012). Research suggests that these relational difficulties may contribute to the development and maintenance of ED symptoms, as individuals seek alternative means to fulfill unmet social needs (Dufresne et al. 2020). In this context, eating control and compulsive behaviors may serve as maladaptive coping mechanisms, providing a sense of stability in the absence of secure interpersonal bonds. This interplay underscores the intrinsic relationship between personality and ED symptoms, where detachment‐related traits could perpetuate a cycle in which ED behaviors replace interpersonal fulfillment. To target detachment in individuals with AN, clinical interventions should focus on enhancing social engagement, trust, and emotional expression, while addressing underlying interpersonal fears in the therapeutic relationship as well as in their lives.

The DSM‐5 defines the five major personality domains as distinct and well‐defined categories (American Psychiatric Association 2013). Our network analysis revealed that, among these five domains, traits supporting three of these domains are more central to the clinical presentation of AN in youth. More importantly, our findings highlight the complex interdependencies between these domains, suggesting that their interactions—rather than any single trait in isolation—may play a key role in the development and maintenance of the disorder. For instance, the negative affectivity domain was strongly linked to detachment. Submissive traits—associated with negative affectivity—and depressive symptoms were closely connected to traits from the detachment domain such as self‐demeaning, dolefulness, inhibition, and personal alienation. Furthermore, certain detachment‐related traits (e.g., personal alienation and self‐demeaning/dolefulness) were closely linked with asceticism, a trait within the conscientiousness domain, which itself was proximal to the drive for thinness node. These findings underscore the intricate interplay among personality traits, suggesting that the drive for thinness, restrictive eating behaviors, and depressive symptoms may be underpinned by a broader, interconnected personality structure. Taken together, our results support the importance for patients of translating anorexic behaviors, depressive symptoms and the focus on thinness into emotional and relational needs that are more closely connected to their lived experiences. Intervention should include helping adolescents express their needs, desires, and opinions both in therapy and in their daily lives. Working on assertiveness, communication skills, and conflict resolution could help them navigate interpersonal relationships more effectively, fostering healthier social interactions. In addition, enhancing self‐awareness and emotional regulation may support individuals in better managing their emotions and relationships. Radically Open Dialectical Behavior Therapy (RO DBT) could represent a particularly promising treatment approach. RO DBT targets maladaptive overcontrol by promoting social connectedness, emotional expression, and cognitive flexibility, dimensions that align with the personality traits identified in our study. Empirical studies in both adult and adolescent populations with AN have reported significant improvements not only in eating disorder symptoms, but also in psychological flexibility and overcontrolled personality traits (e.g., Baudinet et al. 2022; Isaksson et al. 2021). Further studies are needed to better understand how intervening in any of these features might influence the clinical profile of adolescents with AN, and their recovery.

This study has some limitations. First, the predominance of female participants and the scarcity of individuals with binge‐eating/purging AN (AN‐BP) subtype in our sample precluded the assessment of potential moderating effects of gender or AN subtype (AN‐R vs. AN‐BP). Thus, future studies with larger and more diverse pediatric samples are warranted to address this gap. Second, the cross‐sectional nature of the study does not allow the inference of cause‐and‐effect relationships. Consequently, longitudinal and experimental studies would provide valuable insights into the dynamic relationships between eating symptoms, comorbid symptoms and personality traits in adolescents with AN. Third, the generalizability of our results may be limited by some methodological factors, particularly the fact that our sample consisted exclusively of adolescents who had received specialized care. This raises the question of whether these individuals exhibit traits that differ from those of adolescents with AN in the broader community. Finally, relying solely on self‐report measures for assessing eating symptoms, comorbid symptoms, and personality traits may introduce bias, as some adolescents might have exaggerated or minimized their symptoms and traits. Employing a combination of self‐reported questionnaires and clinical interviews could provide a more objective and comprehensive assessment.

## 6 Conclusion

There is a well‐established need to better delineate the psychopathology of AN and to identify psychological traits that may impact patients’ clinical profiles and treatment outcomes (Martinez and Craighead 2015; Westen and Harnden‐Fischer 2001). Integrating personality traits into the identification of key symptoms in AN could allow clinicians to develop treatments that address both eating and comorbid psychiatric symptoms while tailoring interventions to individual personality characteristics. Our findings indicate that, in addition to the drive for thinness and depressive symptoms, traits from the DSM‐5 domains of conscientiousness, negative affectivity, and detachment may also contribute to the clinical presentation of AN in adolescents at the time of admission to specialized ED services. These results align with existing literature and represent a valuable advancement towards a more comprehensive approach to personality in ED. Continued research, particularly longitudinal and experimental studies, is essential to enhance our understanding of how these traits contribute to the development and persistence of eating pathology.

## Ethics Statement

This study has been approved by the ethics committee of the coordinating center (CHU de Québec‐Université Laval: # MP‐20‐2015‐2323) and by the Ethic Committees of each participating center.

## Consent

Before participating in this study, all adolescents provided their written informed assent, in addition to their parents/legal guardians written informed consent.

## Conflicts of Interest

The authors declare no conflicts of interest.

## Supporting information

Supporting Information.

## Data Availability

The data that support the findings of this study are available on request from the corresponding author. The data are not publicly available due to privacy or ethical restrictions.
