# Supplementary material for: Examining Clinical Symptoms and Personological Traits in Adolescents With Anorexia Nervosa: A Network Analysis Approach
Source: J Clin Psychol. 2025 Jul 19;81(11):1070–80. doi: 10.1002/jclp.70018 (PMC12501823; doi:10.1002/jclp.70018)
Supplement: Supplementary file 1 — Supporting Information. [file JCLP-81-1070-s001.doc]

**Supporting Information**

**Table S1.** Centrality indices estimates of the Network.

Unr, unruly; Sub, submissive; PeA, personal alienation; PCA, doleful and self-demeaning; P, perfectionism; Opp, oppositional; MF, maturity fear; MAS, Multidimensional Anxiety Scale for Children; LSE, low selfesteem; Int, introversive; Inh, inhibited; InA, interpersonal alienation; IN, interpersonal insecurity; ID, interoceptive deficits; For, forceful; Ego, Egotistic; ED, emotional dysregulation; DT, drive for thinness; Dra, dramatizing; Con, conforming; CDI, children's depression inventory; BU, bulimia; Bor, bordeline; BD, body dissatisfaction; ASC, ascetism.

|  | Strength | Betweenness | Closeness |
| --- | --- | --- | --- |
| Unr | 1.497 | 22 | 0.004 |
| Sub | 2.010 | 66 | 0.004 |
| PeA | 1.239 | 58 | 0.004 |
| PCA | 1.627 | 54 | 0.004 |
| P | 1.116 | 22 | 0.003 |
| Opp | 1.297 | 16 | 0.004 |
| MF | 0.613 | 0 | 0.002 |
| MAS | 1.105 | 4 | 0.003 |
| LSE | 1.121 | 36 | 0.003 |
| Int | 1.164 | 0 | 0.003 |
| Inh | 1.559 | 56 | 0.004 |
| InA | 0.943 | 12 | 0.003 |
| IN | 1.139 | 20 | 0.003 |
| ID | 0.952 | 18 | 0.003 |
| For | 1.407 | 16 | 0.004 |
| Ego | 1.546 | 58 | 0.004 |
| ED | 1.152 | 14 | 0.003 |
| DT | 1.315 | 24 | 0.003 |
| Dra | 1.469 | 58 | 0.004 |
| Con | 1.666 | 50 | 0.004 |
| CDI | 1.300 | 16 | 0.004 |
| BU | 0.687 | 0 | 0.002 |
| Bor | 1.347 | 2 | 0.003 |
| BD | 1.145 | 28 | 0.003 |
| ASC | 1.178 | 32 | 0.004 |

**Table S2.** The correlation matrix of the variables included in the network.

Unr, unruly; Sub, submissive; PeA, personal alienation; PCA, doleful and self-demeaning; P, perfectionism; Opp, oppositional; MF, maturity fear; MAS, Multidimensional Anxiety Scale for Children; LSE, low selfesteem; Int, introversive; Inh, inhibited; InA, interpersonal alienation; IN, interpersonal insecurity; ID, interoceptive deficits; For, forceful; Ego, Egotistic; ED, emotional dysregulation; DT, drive for thinness; Dra, dramatizing; Con, conforming; CDI, children's depression inventory; BU, bulimia; Bor, bordeline; BD, body dissatisfaction; ASC, ascetism.

**Figure S1.** Average correlation between centrality indices of the networks sampled with persons dropped and the original sample. Lines indicate the means and areas indicate the range from the 2.5th quantile to the 97.5th quantile.

**
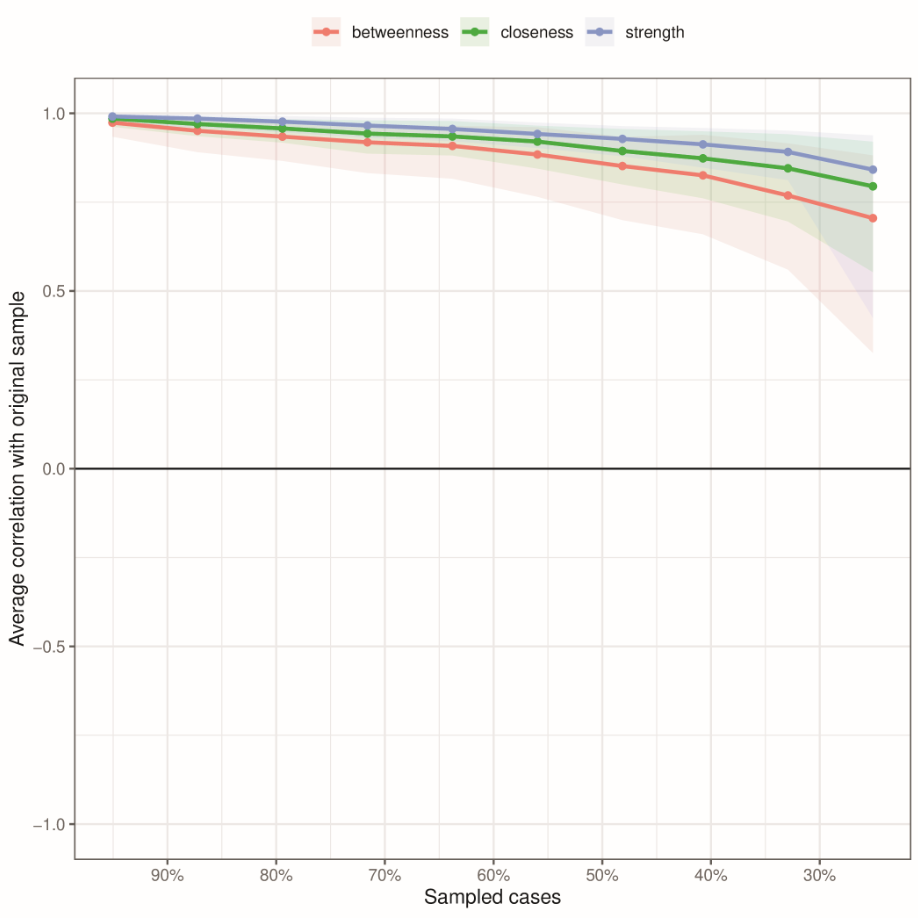
**

**Figure S2.** Bootstrapped confidence intervals of estimated edge-weights of the network.


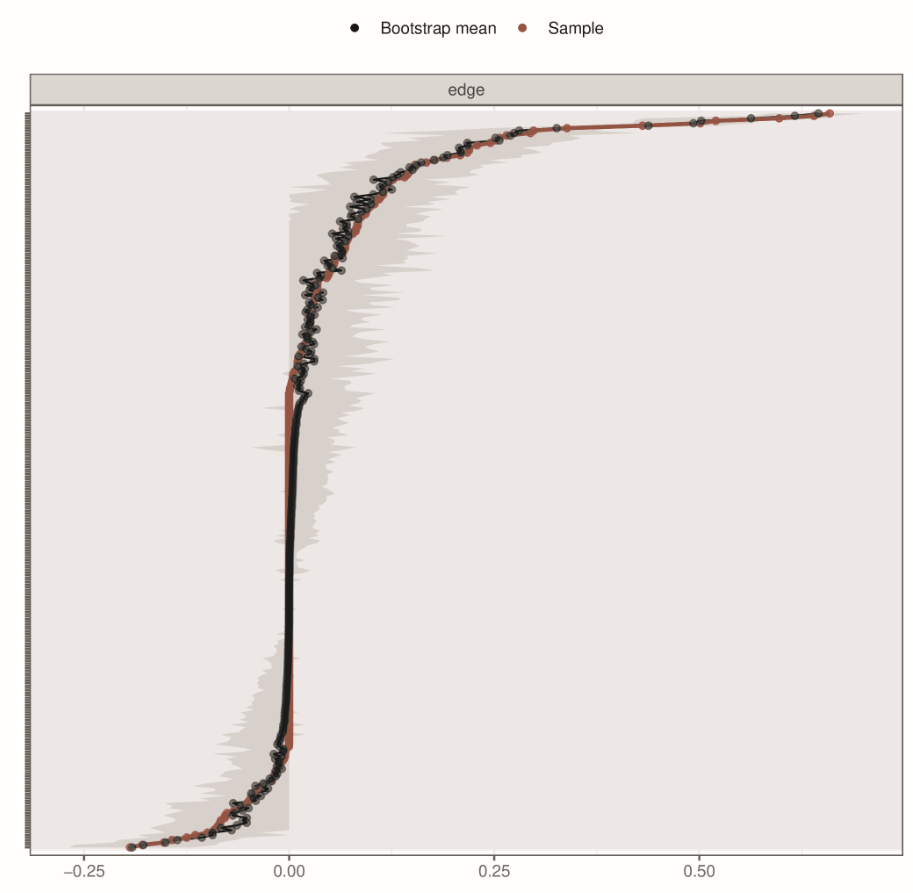


**Code used for network analyses with RStudio Version 4.3.0 in the present paper.**

library("readxl")

library("qgraph")

library("bootnet")

library("reshape2")

library("ggplot2")

library("lavaan")

library("lme4")

library("glmnet")

library("depmixS4")

library("huge")

library("BayesFactor")

library("ltm")

library("ega")

library("psych")

library("networktools")

data <- read_xlsx("/Users/admin/Desktop/Classeur.xlsx")

Names <- names(data)

names(data) <- c ("Int", "Inh", "Dol", "Sub","Dra", "Ego", "Unr", "For", "Con","Opp","Dem","Bor",

"MASC","CDI", "DT", "BU", "BD", "LSE", "PeA", "IN", "InA", "ID", "ED", "P", "ASC","MF")

data <- as.matrix(data)

data <- matrix(as.numeric(data), ncol = length(Names))

datomit <- na.omit(data)

colnames(data) <- colnames(datomit) <-c ("Int", "Inh", "Dol", "Sub","Dra", "Ego", "Unr", "For", "Con","Opp","Dem","Bor","MASC","CDI", "Dt", "BU", "BD", "LSE", "PeA", "IN", "InA", "ID", "ED", "P", "ASC", "MF")

#Applying Goldbricker Function to identify redundant nodes

gb <- goldbricker(datomit, p = 0.05, method = "hittner2003", threshold = 0.25,

corMin = 0.5, progressbar = TRUE)

reduced_data <- net_reduce(data = datomit, badpairs = gb, method = "PCA")

reduced_data_final = reduced_data[,-ncol(reduced_data)]

reduced_data_final = cbind(reduced_data_final,datomit[,25])

reduced_data_final = cbind(reduced_data_final,datomit[,22])

colnames(reduced_data_final)[24] = "ASC"

colnames(reduced_data_final)[25] = "ID"

save(reduced_data_final,file = "reduced_data_final.RData")

NAlocation <- apply(data, 1, function(x) any(is.na(x)))

Datafinal = data[,-3] #removing column Dol

Datafinal = Datafinal[,-10] #removing column Dem

Names<-Names[-3]

Names<-Names[-10]

Names[25]<-"MACI.PCA.DOL.DEM"

Datafinal = cbind(Datafinal,rep(NA,length(NAlocation))) #adding column to merge Dol and Dem variables

colnames(Datafinal)[25] = "PCA.DOL.DEM"

j=1

for (i in 1:length(NAlocation)){

if (NAlocation[i]==FALSE){

Datafinal[i,"PCA.DOL.DEM"]= reduced_data_final[j,23]

j=j+1

}

}

data = Datafinal

corMat = cor(data, use="pairwise.complete.obs")

pdf("/Users/admin/Desktop/Analyse/reseau.pdf", width = 9, height = 7)

Graph <- qgraph(corMat, graph = "glasso", layout =

"spring", tuning = 0.5, sampleSize = nrow(data), cut =

NULL ,minimum = 0, maximum = 1, details = FALSE,

esize = 20, lambda.min.ratio = 0.01, groups = list

("MACI"=c(1:10,25), "MASC" = 11, "CDI"= 12, "EDI"= 13:24), nodeNames = Names, legend.cex = 0.25,

vsize = 5, esize = 15, pastel = TRUE, posCol = "#003399",

negCol = "#FF9933",color = c("red", "yellow", "pink",

"orange", "green"), borders = FALSE, vTrans = 200)

dev.off()

cent <- centrality(Graph)

cent$OutDegree

cent$Closeness

cent$Betweenness

pdf("/Users/admin/Desktop/Analyse/centrality.pdf", width = 7, height = 7)

centralityPlot(Graph,include = c("Strength","Betweenness","Closeness"),scale="z-scores")

dev.off()

graph <- estimateNetwork(data, default = "EBICglasso", tuning = 0.5, corMethod = "npn")

boot1 <- bootnet(graph, nCores = 8, nBoots = 2500, type = "nonparametric")

pdf("/Users/admin/Desktop/Analyse/boot1.pdf", width = 7, height = 7)

plot(boot1, order = "sample", labels = FALSE)

dev.off()

boot2 <- bootnet(graph, nCores = 8, nBoots = 2500, type =

"case", statistics = c( "strength", "closeness","betweenness"))

pdf("/Users/admin/Desktop/Analyse/boot2.pdf", width = 7, height = 7)

plot(boot2, statistics = "all")

dev.off()

corStability(boot2)
